# Nigella Sativa’s Anti-Inflammatory and Antioxidative Effects in Experimental Inflammation

**DOI:** 10.3390/antiox9100921

**Published:** 2020-09-26

**Authors:** Raluca Maria Pop, Octavia Sabin, Șoimița Suciu, Stefan Cristian Vesa, Sonia Ancuța Socaci, Veronica Sanda Chedea, Ioana Corina Bocsan, Anca Dana Buzoianu

**Affiliations:** 1 Department of Pharmacology, Toxicology and Clinical Pharmacology, Iuliu Haţieganu University of Medicine and Pharmacy Cluj-Napoca, 400337 Cluj-Napoca, Romania; raluca_parlog@yahoo.com (R.M.P.); stefanvesa@gmail.com (S.C.V.); bocsan.corina@umfcluj.ro (I.C.B.); abuzoianu@umfcluj.ro (A.D.B.); 2 Department of Physiology, Iuliu Haţieganu University of Medicine and Pharmacy Cluj-Napoca, 400337 Cluj-Napoca, Romania; ssuciu@yahoo.com; 3 Department of Food Science, University of Agricultural Sciences and Veterinary Medicine of Cluj-Napoca, Calea Mănăştur 3-5, 400372 Cluj-Napoca, Romania; sonia.socaci@usamvcluj.ro; 4 Research Station for Viticulture and Enology, 515400 Blaj, Romania; chendeaveronica@yahoo.com

**Keywords:** Ranunculaceae, Nigella sativa oil, anti-inflammatory effect, antioxidant effect, antinociceptive effect

## Abstract

*Nigella sativa* (NS) has been used for centuries in various inflammatory conditions because of its anti-inflammatory and antioxidant activities. The study aimed to evaluate the anti-inflammatory, antinociceptive and antioxidant activity of Nigella sativa oil (NSO) in two models of acute (carrageenan-induced) and sub-acute inflammation (complete Freund’s adjuvant induced) in rats. Materials and Methods: NSO was administered orally 1, 2 and 4 mL/kg in the acute phase. For subacute phase, NSO was administered 4 mL/kg, 7 days before or after inflammation induction, or in association with diclofenac 5 mg/kg. Results: The gas chromatography coupled with mass spectroscopy (GC-MS) analysis showed that NSO is an important source of bioactive compounds, especially p-cymene and thymoquinone. In the acute phase, 1.5 h after administration, NSO (2 and 4 mL/kg) determined an anti-inflammatory effect comparable with that of diclofenac. In the sub-acute administration, NSO had no anti-inflammatory effect. The analgesic effect of NSO was observed only in the sub-acute inflammation in the analgesy-meter test. NSO as treatment proved its antioxidant effect through the reduction of malondialdehyde (MDA) and oxidized glutathione (GSSG), and increases in hydrogen donor capacity (DH) compared to the control group, but the effect was not as intense as that of diclofenac. Conclusion: The present study has proven inconstant anti-inflammatory, analgesic and antioxidative properties of NSO.

## 1. Introduction

Inflammation is a primary physiological mechanism, a normal defense mechanism that aims to help the organism to fight against initial injury such as that caused by allergens, microorganisms, or mechanical or chemical injury and its consequences, including pain [1,2]. An uncontrolled inflammatory process can induce prolonged chronic inflammation, which, most of the time, acts as an etiologic factor in the development of different chronic diseases [3,4,5,6]. Furthermore, during chronic inflammation processes, oxidants (ROS) are produced excessively, leading to oxidative stress [7]. At some point, inflammation and oxidative stress can induce each other [8], therefore, it is important to follow these tightly interconnected processes. This idea represents the basis from which the antioxidants stand as potential disruptors of chronic inflammatory processes.

Through their wide spectrum, inflammatory diseases are receiving an increasing interest in society throughout the world, and patients are becoming more aware on their importance and effect on their lives. Currently, both nonsteroidal anti-inflammatory drugs (NSAIDs) and corticosteroids are used in the treatment of inflammatory conditions [1]. They are essential therapeutic tools, but the concerns related to long-term toxicity and adverse reactions represent strong limitations to their use.

In this context, there is a growing interest to find alternative treatments in plants with bioactive compounds that can fight against inflammation, switching from traditional ethnobotanical studies to those that are more systematically pharmacological. Thus, areas of the research concerning plant efficacy, safety and their mechanisms of action are now trying to find safe alternatives in the treatment of inflammatory conditions.

*Nigella sativa* (NS) has been used for centuries to treat or to prevent different pathologies like cardiovascular diseases, gastrointestinal disorders, respiratory diseases, hypertension, dyslipidemia, diabetes, or various types of cancer [9]. *Nigella sativa* belongs to Ranunculaceae family. It is an annual plant which grows in southern Europe or Asia, especially in Syria, India, Pakistan, Turkey and Saudi Arabia [9]. The beneficial effects empirically observed in various diseases are supposedly explained by its rich composition in bioactive compounds. The most important compounds in NS and NS oil (NSO) have already been identified and described in the literature; their pharmacological effects are partially explained in animal and human studies. These components are fatty acids, vitamins, minerals, phytosterols, monoterpene compounds like p-cymene, α-thujene, carvacrol, α- and β- pinene, and thymoquinone [10,11,12,13].

Numerous in vivo and in vitro studies mention the synergistic effect of NS compounds [14,15], further translated in their anti-inflammatory, analgesic, and antioxidant activity, as well as their anti-hyperlipidemic; anti-cancer; anti-diabetic; cardiovascular-protective; gastro-protective; and hepato-, neuro-, or immuno-protective activities [16,17,18,19,20].

The aim of this study was to evaluate the anti-inflammatory, antinociceptive, and antioxidant activity of NSO using two models of acute and subacute inflammation in rats. Since inflammatory processes are accompanied by pain most of the time, the associated antinociceptive effects of NSO oil were also investigated.

## 2. Materials and Methods

### 2.1. Chemicals

Complete Freund’s adjuvant (CFA) and carrageenan were purchased from Sigma-Aldrich (St. Louis, MO, USA). Sodium diclofenac solution, physiological serum, and cold-pressed Nigella sativa oil (NSO) were purchased from a local pharmacy. Thymoquinone and thymol standards were purchased from Sigma-Aldrich (St. Louis, MO, USA).

### 2.2. Gas Chromatography Coupled with Mass Spectroscopy (GC-MS) Analysis of Nigella Sativa Iil

The analysis of volatile compounds was carried out on a GCMS QP-2010 (Shimadzu Scientific Instruments, Kyoto, Japan) model gas chromatograph–mass spectrometer. Oil solution in hexane (1% *v*/*v*) was used for injection. The volatile compounds were separated on a Zebron ZB-5ms capillary column (30 m × 0.25 mm i.d and 0.25 μm film thickness). Helium (1 mL/min) was used as a carrier gas, and the split ratio was 1:50. The following temperature program was used for the column oven: from 50 °C (held for 2 min) to 160 °C at 4 °C/min, then further heated to 250 °C with 15 °C/min increments and held for 10 min. The injector, ion-source and interface temperatures were set at 250 °C. The electron impact (EI) MS mode was used, and the ionization energy of 70 eV and operating in the full scan (mass range scanned 40–500 *m*/*z*) were applied.

The volatile compounds were tentatively identified by comparing the mass spectrometric information of each separated peak with those from NIST27 and NIST147 mass spectra libraries (considering a minimum similarity of 85%) and also by comparison with retention indices drawn from www.pherobase.com [21] or www.flavornet.org [22] (for columns with a similar stationary phase to the ZB-5ms column). The relative percentage of each compound was estimated as a fraction of its integrated ion area from the total ion chromatograms (TIC) area (100%). In the case of standard compounds thymoquinone and thymol, calibration curves were drawn by injecting solutions (using the same conditions as for the sample) of different concentrations (0.01–1 mg/mL).

### 2.3. Animals

Fifty white female rats (Wistar-Bratislava) were weighted, randomized and divided into five groups. The rats with body weights between 200 and 250 g were kept at the Biobase of Faculty of Medicine, “Iuliu Haţieganu” University of Medicine and Pharmacy Cluj-Napoca. During the experiment, standard conditions (25 °C, 50 ± 15% humidity, natural light–dark cycle, standard pellets, and water ad libitum) were provided. The protocols were approved by the University of Medicine and Pharmacy “Iuliu Haţieganu” Ethics Committee (57/03.02.2017) and by the Sanitary-Veterinary and Food Safety Directorate from Cluj-Napoca (60/08.05.2017). The national and international guidelines referring to the animals’ care and use were followed. To avoid the use of an excessive number of rats in the experimental study, rats were used in both acute and chronic inflammation induced models. The carrageenan-induced acute inflammatory process was firstly induced, followed by the FCA chronic inflammatory process after a wash-up period of 2 weeks. Between experiments, a two weeks recovery period was imposed.

### 2.4. Carrageenan-Induced Hind Paw Edema in Rats

The acute inflammatory process was induced using a 1% carrageenan solution (dissolved in normal saline solution). Each rat was injected with 0.1 mL carrageenan solution in the left hind rat paw (sub plantar region). The groups are described in Table 1. Diclofenac sodium and NSO dosage were established according to the previously reported results [23].

### 2.5. Inflammatory Edema Assessment

A digital plethysmometer (Ugo-Basile, Milan, Italy) was used to measure the paw edema by recording the volume of fluid displaced by the affected paw. The results were expressed as paw volume. Moreover, the calculation of the percentage inhibition of paw volume was performed using the mean difference of the paw volume measured in the control groups and the one measured in the treated groups.

(1) $$\frac{\mathrm{Vc}-\mathrm{Vt}}{\mathrm{Vc}}\;\times \;100$$

where Vc = CTRL group paw volume, and Vt = treated groups’ (CTRL+, NSO_1, NSO_2, NSO_3) paw volume.

### 2.6. Mechanical Nociceptive Response Measurement

An analgesy-meter (Ugo Basile, Milan, Italy) was used for the paw pressure test. Plantar mechanical pressure was applied linearly by increasing the mechanical force to the rat’s paw. The retraction of the paw of the rat’s squeak was recorded as the latency response. The value of 500 g was set as cut-off pressure [24].

### 2.7. Time Point Measurements

The measurements were performed initially 24 h before carrageenan injection and at 90, 120, 180, and 360 min after carrageenan injection. The drugs were administered immediately after carrageenan injection.

### 2.8. Freund’s Adjuvant (FA)-Induced Hind Paw Edema in Rats

The sub-acute inflammatory process was induced using FA injection. Each rat was injected with 0.1 mL FA solution in the left hind rat paw (sub plantar region). The groups are described in Table 2. NSO dosage was established based on the results obtained from the carrageenan-induced inflammatory process, as described above (see Section 2.5).

### 2.9. Inflammation and Nociceptive Response Measurements

A hot–cold plate (Ugo Basile, Milan, Italy) was used to evaluate the central and peripheral pain mechanisms. Heat (50 ± 0.1 °C) hypersensitivity was determined by placing each rat on the metal plate. The nociceptive withdrawal response was considered at the first sign of paw licking or jumping. Tissue damage was prevented by setting a 60 s cut-off time value [24]. The mechanical nociceptive response measurement and the inflammation edema assessment were also performed as described above (see Section 2.6).

### 2.10. Time Point Measurements

The measurements were performed initially 24 h before FA injection and at 1 day, 3 days, 7 days after it.

### 2.11. Oxidative Stress Analysis

At the beginning of the experimental period, blood samples were collected using a thick capillary from the tail vein, while at the end of the experiment, blood samples were collected from the retro bulbar venous sinus on the anticoagulant collection tube for serum preparation. The serum samples obtained after centrifugation were stored at −80 °C until they were analyzed. The serum oxidative stress status was evaluated using a spectrophotometer as previously described by Conti et al. [25] for malondialdehyde (MDA), Hu [26] for glutathione (GSH), Vats et al. [27] for oxidized glutathione (GSSG), GSH/GSSG ratio, Janaszewska and Bartosz [28] for hydrogen donor capacity (DH), and Flohé and Otting [29] for superoxide dismutase (SOD).

### 2.12. Statistical Analysis

The statistical analysis was performed using the Medcalc 12.5 program. The used data were considered as quantitative variables. A Kolmogorov–Smirnov test was used to check the normality of continuous variables. The characterization of quantitative data was performed using the mean and standard deviation. Measurement of mean statistical difference at various moments of the experiment was performed using the ANOVA test for repeated measurements. The statistical significance was considered for *p* < 0.05.

## 3. Results

### 3.1. GS-MS Analysis of Nigella Sativa Oil 

Thymoquinone (TQ) and thymol concentrations, the main compounds of NSO with an anti-inflammatory effect, were 0.375 (mg/mL) and 0.021 (mg/mL), respectively. The chemical composition of the volatile compounds identified in NSO is presented in Table 3.

As observed, in the volatile fraction of NSO, 13 compounds were identified, and, among them, p-cymene was the major compound. The monoterpene hydrocarbons group represented by α−thujene, α−pinene, 4(10)−thujene (sabinene), β−pinene, p-cymene, D-limonene and sabinene hydrate had the highest percentage (62.97%) among identified compounds. The monoterpenoid ketones, represented by TQ, were the next predominant group, with 29.85%. The other identified compounds were found in a lower percentage (7.18%) as follows: sesquiterpene with 3.72%, terpene phenols with 2.35%, monoterpenoid alcohols with 0.35% and cycloalkenes (0.76%).

### 3.2. Paw Edema in Acute Inflammation

All animals presented a marked unilateral peripheral paw edema after carrageenan injection. The measured paw volume in the animal groups presented a progressive increase, reaching the maximum values at 4.5 h time point after carrageenan injection. Table 4 presents the animal groups’ edema time course and NSO inhibition percentage. The group treated with sodium diclofenac had the highest inhibition percentage when compared with groups treated with NSO, reaching the maximum level of inhibition (33%) after 3 h. Among NSO groups, those treated with 4 mL/ kg BW reached the highest inhibition effect (23.41%) after 6 h. Overall, the inhibitory effect of NSO in all three doses was significant in comparison to the control group, starting from the 90 min time-point. However, the effect lasted only 3 h for the low dose of NSO, while it was longer for the other two doses. The effect was similar to that of diclofenac at 270 and 360 min, respectively, for NSO_2 and NSO_4 (*p* > 0.05). The group fed with the lowest dose of oil (NSO_1) reached the significantly inhibitory effect after 1.5 h from the induction of the inflammatory process, and it lasted maximal 3 h.

### 3.3. Antinociceptive Effect of NSO on Acute Inflammation

The withdrawal threshold values obtained during the mechanical test were statistically different between groups at all-time point, except baseline (0 min). The positive control group was also statistically different as compared to the other groups, with higher threshold values, indicating the diclofenac analgesic effect. NSO had no analgesic effect on carrageenan-induced inflammation in rats (Table 5).

### 3.4. Paw Edema in Sub-Acute Inflammation

Diclofenac sodium (10 mg/kg) treatment showed a significant decrease in FA-injected paw edema volume after 24 h and 168 h (*p* < 0.05) as compared to the control group (Table 6). The groups treated only with NSO, as prevention or as treatment, were not statistically different when compared with the control group (Table 6). The group treated with both sodium diclofenac and NSO had significantly lower values than the control group 72 and 168 h after treatment administration (Table 6). Furthermore, the NSO_Adj group was not statistically different when compared with the CTRL+ group (Table 6).

### 3.5. Antinociceptive Effect of NSO on Sub-Acute Inflammation—Mechanical Hyperalgesia Evaluation

The antinociceptive effects of NSO in the mechanical test can be demonstrated by increased withdrawal threshold values when compared with the control group (Table 7).

In the FA-induced sub-acute inflammation model, the positive control group had higher values than the CTRL group at all time point measurements but reached statistical significance only after 7 days of treatment. The analgesic effect of NSO (4 mL/ kg BW) was also observed after 7 days of treatment when administrated for both preventive and treatment purposes (Table 7).

Moreover, at this time point, the treatment group had the same effect as the diclofenac group (*p* < 0.05), indicating a strong analgesic effect (Table 7). The highest withdrawal threshold values, statistically different when compared with the CTRL group, were obtained for the NSO_Adj group (Table 7). The same analgesic effect as sodium diclofenac (5 mg/kg BW) was obtained after 1 and 3 days of treatment when the threshold values were not statistically different when compared with the positive CTRL group (Table 7). Moreover, after 7 days of treatment, the NSO_Adj group had a higher pain threshold (10.6 ± 1.34) when compared to the CTRL+ group (8.5 ± 1.43) (*p* = 0,034) (Table 7).

### 3.6. Antinociceptive Effect of NSO on Sub-Acute Inflammation—Thermal Hyperalgesia Evaluation

The antinociceptive effects of NSO in hot plate test can be demonstrated by an increased time response as compared with the control group (Table 8). It can be observed that one hour after treatment administration, the NSO preventive administration had a strong analgesic effect with statistically different values when compared with CTRL group and with no statistically significant differences when compared with the CTRL+ group (Table 8). The same results were obtained for the NSO_Adj group (Table 8). After 24 h, NSO (4 mL/kg BW) lost its analgesic effect on the hot plate test. NSO showed a significant analgesic effect when it was administrated as adjuvant treatment in combination with sodium diclofenac (Table 8).

### 3.7. Effect of NSO on Oxidative Stress in Sub-Acute Inflammation

**Figure 1:**
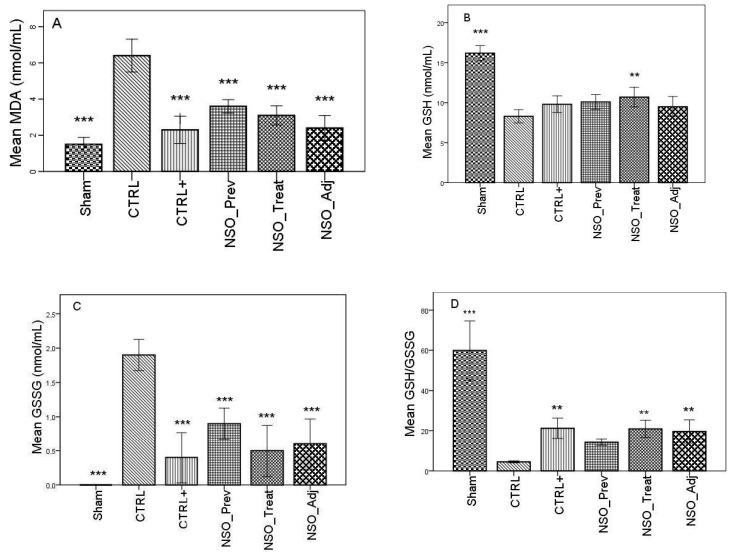

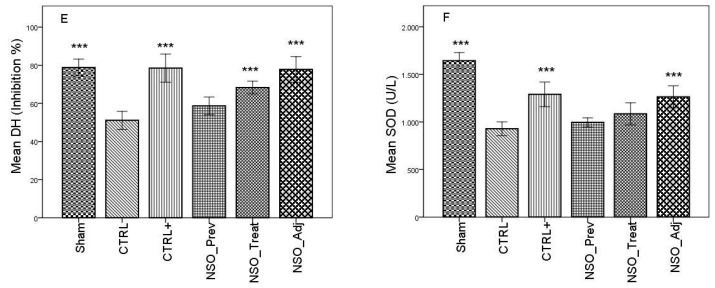
*Nigella sativa* oil treatment prevents FA-induced oxidative stress in serum. MDA concentrations (**A**), glutathione (GSH) concentration (**B**), oxidized glutathione (GSSG) concentration (**C**), GSH/GSSG ratio (**D**), hydrogen donor capacity (DH) inhibition percent (**E**) and, superoxide dismutase (SOD) activities (**F**) in rats serum. Data are presented as means ± SD (*n* = 10 for each group). ** *p* < 0.01 and *** *p* < 0.001 vs. control group. For comparison between groups, one-way analysis of variance (ANOVA) with Tukey multiple comparison tests was used.

FA induced considerable inflammation, increasing the levels of MDA and GSSG and decreasing the levels of GSH, DH and SOD in the serum of the sensitized group when compared with the Sham group. The group treated with sodium diclofenac (CTRL+) had a positive effect on improving these parameters, especially in the case of MDA, GSSG and DH, where the values were not statistically different than those identified in the Sham group. Moreover, the NSO administration resulted in a significant improvement in all of these parameters. Specifically, in the case of MDA, there was no statistically significant difference between the CTRL+ group and the NSO_Treat and NSO_Adj groups (Figure 1A). In the case of GSH and GSSG (Figure 1B–D), the NSO had the same effect as diclofenac sodium. No statistically significant difference was found between the CTRL+ and NSO groups. Concerning DH and SOD analysis (Figure 1E,F), NSO had an inconstant effect. NSO as treatment restored significantly the activity of DH, but not of SOD when compared to the control group. The intensity of the effect was lower compared to diclofenac. NSO as adjuvant restored the activity of both enzymes in a similar manner to diclofenac monotherapy.

## 4. Discussion

The present study demonstrated the anti-inflammatory effect of NSO in acute inflammation. The anti-inflammatory effects of NSO are comparable with those induced by diclofenac. The analgesic effect of NSO was observed only in the sub-acute inflammation model when the mechanical analgesia was evaluated. In addition, NSO added to diclofenac had a tendency to increase the analgesic and anti-inflammatory effects of diclofenac in sub-acute inflammation, but the differences did not reach the level of statistical significance. The analgesic and anti-inflammatory effects of NSO treatment were related to its antioxidative action, demonstrated by the reduction of MDA and GSSG and increases in DH.

The present study offers also valuable information regarding the composition of NS oil. The results of the present study showed that NSO is an important source of bioactive compounds, especially p-cymene, TQ and α-thujene. The percentages of the identified compound are consistent with other reported results [30,31].

Diclofenac is an NSAID with known anti-inflammatory and analgesic properties due to the inhibition of cyclo-oxygenase 2 COX2 and prostaglandins synthesis [23]. Diclofenac significantly reduced the paw edema in both carrageenan and FA induced inflammations, and it reduced the pain perception after mechanical or thermal stimuli in both acute and sub-acute settings [32].

NSO in all three concentrations reduced paw edema in carrageenan induced inflammation. The intermediate (2 mL/kg) and high (4 mL/kg) doses determined a significant reduction of paw edema starting from 1.5 h time point, while in the case of the minimum 1 mL/kg dose, the anti-inflammatory effect was noticed only at 1.5 and 3 h time points when compared with the control. Even though a significant inhibition of paw edema was achieved at all time points (notably, however, one that was less intense than diclofenac within the first 4.5 h), a constant increase in paw edema was observed. After this period, a decline was observed as compared with the control group. The maximal anti-inflammatory effect was noticed at 6 h time point for NSO 4 mL/kg when the paw volume inhibition was not statistically different as compared with sodium diclofenac. Thus, the anti-inflammatory effects of orally administrated NSO 4 mL/kg are comparable with 5 mg/kg diclofenac.

Similar results were reported in previously published studies. In the study of Al-Ghamdi et al., NS volatile oil and NS aqueous extract orally administered reduced significantly the paw edema after 3 h in a similar model of carrageenan induced inflammation, but the anti-inflammatory effect was less intense than that produced by indomethacin [33]. Pise et al. reported a significant anti-inflammatory effect of NS oil demonstrated by reduction of paw edema at the 3 h time point compared to the control group, but it was less intense than aspirin’s anti-inflammatory effect [1]. The fact that, in our study, a more significant decrease in edema is obtained after 4.5 h, 1,5 h later than that reported by the study of Pise et al., can be explained by the used NSO doses, which were higher than those administered in the present study. Therefore, we can assume that the anti-inflammatory effect of NSO is dose-dependent, time-dependent and it might involve several mechanisms.

NSO in continuous administration (as preventive treatment) or after sub-acute inflammation induction (as therapy) did not produce a significant anti-inflammatory effect. The results are similar to the data reported by Nasuti et al. The authors did not report a significant difference of paw edema after 5 days of treatment in FA-induced arthritis. They observed an anti-inflammatory effect of NSO in a higher dose, but not in low one in a model of FA-induced arthritis after 25 days of treatment. The effect was less intense than that of indomethacin [34]. These observations might confirm that NS has an anti-inflammatory effect that is less potent than NSAIDs, and in a chronic model of arthritis, a longer duration of administration is required to validate it.

One of the cardinal signs of inflammatory states is that normally innocuous stimuli produce pain [35]. Inflammatory mediators released in both acute and chronic processes interact with neurons to produce hypersensitivity. Diclofenac sodium reduced the pain perception after mechanical or thermal stimuli in both acute and sub-acute settings.

In the carrageenan-induced inflammation, the authors used the Randall–Selitto test, one of the methods that evaluate mechanical hyperalgesia [36]. NSO in single administration did not alter the pain level in carrageenan-induced inflammation, so the authors confirm that NSO did not induce a central analgesic effect in acute inflammation. Diclofenac inhibiting prostaglandin synthesis in the periphery reduces the inflammation near tissue lesions and attenuates central nervous stimulation. It might have a central analgesic effect [36]. In the present study, diclofenac increased the pain level in all time points when compared to the control group, while NSO reduced the pain threshold, especially at the 3 and 6 h time points. This may suggest that diclofenac validated both its peripheral and central analgesic effects, while for NSO, other tests should be used to investigate its possible peripheral analgesic effect. The possible analgesic effect of NSO is not correlated with COX system inhibition like in the case of NSAIDs, and other mechanisms should be investigated.

In sub-acute inflammation, the analgesic effect of NS is inconstant. The authors used the analgesy-meter test and the hot/cold plate test, both investigating central analgesia and possible intervention of opioid receptors. NSO in prophylactic administration significantly increased the latency time reaction in the hot plate test 1 h after FA-induced inflammation compared to the control group, but the reaction time declined in the subsequent time points. NSO as add-on therapy to diclofenac increased the latency time in the hot plate test and the pain threshold in analgesy-meter tests. The onset of the effect was different—it started 1 h after administration of FA in the case of the hot plate test, while in the second test, it was noticed after 24 h. The maximal effect was obtained in both cases after 7 days. Combination of NSO and diclofenac showed a superior analgesic effect on the control group or monotherapy with NSO as preventive or curative treatment. These observations suggest that NSO may have an immediate analgesic effect if a thermal stimulus is applied, but it enhances and prolongs the analgesic effect of diclofenac. The immediate effect could be a consequence of the peripheral effect of NSO, while the prolongation of the diclofenac effect is a result of a possible central effect.

Inconsistent results were also reported in previously published research. Nasuti et al. noticed that NSO had antinociceptive properties in a contralateral hind paw but not in a inoculated one after 25 days of treatment [34]. This observation differs from the present study, which did not report the antinociceptive effect in the contralateral paw, but the final evaluation was performed after a shorter duration of therapy.

A more pronounced analgesic effect of NS could be attributed to its constituents’ polyphenols and thymoquinone. Ghannadi et al. showed that polyphenols derived from NS had an immediate but long-lasting analgesic effect, which is dose-dependent, and this effect is not cancelled by pre-administration of naloxone [37] and thus is not produced by the intervention of the opioid system. Similar results were also reported by Hajhashemi et al. for NS and thymoquinone, suggesting that components of NS are responsible for the analgesic effect [38]. However, in a recent study, Abdelgalil et al. raised the hypothesis of a possible analgesic effect of NS that involved also opioid receptors [39]. The secondary analgesia to the anti-inflammatory effect of NS is probably produced by reduction of eicosanoids synthesis (thromboxane A2, prostaglandins and leukotrienes), due to inhibition of COX and lipoxygenase [40].

The NSO as a preventive, curative or add-on therapy reduced oxidative stress in FA-induced inflammation. Similar results were previously reported in the literature. NSO reduced oxidative stress, increasing the activity of antioxidant enzymes, reducing free radicals, like NO and MDA, and inhibiting lipid peroxidation [1,41]. Similar beneficial results were also obtained in the present study. NSO inhibited MDA production and reduced GSSG increase after induction of inflammation, thus reducing oxidative stress. The significantly increased body defense capacity against oxygen reactive species is also related to the GSH/GSSG ratio. GSH, the reduced form, should have a higher concentration than GSSG, the oxidized form [42]. In the present study, NSO treatment raised the GSH/GSSG ratio with almost half of its basal value. NSO_Adj had a similar effect to diclofenac alone. NSO also normalized the antioxidant capacity (DH), but less intensely than diclofenac. The reported results are in accordance with previously published data that showed that NS essential oil normalized the level of enzymes involved in lipid peroxidation (LPX), lactate dehydrogenase (LDH), glutathione (GSH), superoxide dismutase (SOD), glutathione peroxidase (GPx) and catalase (CAT) [43].

The strength of this paper lies in the complex evaluation of anti-inflammatory, analgesic and antioxidative effects of NSO in different models of acute or sub-acute inflammation. The study has some limitation. The authors investigated the effects of entire NS oil, but not the effects of bioactive compounds (TQ, carvacrol, thymol, cymene, 4-terpineol) that may confer particular pharmacological properties. The analgesic and anti-inflammatory effect of NSO in chronic inflammation were investigated after maximal 7 days of treatment, and a longer administration of NSO might increase the intensity and duration of its pharmacological effects.

## 5. Conclusions

NSO in single administration determined an anti-inflammatory effect, which was time dependent. A high dose of NSO had a similar effect to diclofenac. NSO as continuous administration before or after induction of inflammation did not induce an anti-inflammatory effect, nor did it enhance diclofenac’s anti-inflammatory effect. NSO enhances the analgesic effect of diclofenac but has no analgesic effect on single or continuous administration. NSO in monotherapy or added to classical NSAIDs had an antioxidant effect. NSO could represent a therapeutic option in association with classical analgesic–anti-inflammatory medication in acute or chronic inflammation.

## Figures and Tables

**Table 1:** Experimental design of carrageenan-induced acute inflammation.

Groups/Abbrev.	Administrated Substances/Dose	Route
Group I—control group (CTRL)	Normal saline solution	p.o.
Group II—positive control group (CTRL+)	Diclofenac sodium (5 mg/kg BW)	i.p.
Group III—(NSO_1)	NSO (1 mL/ kg BW)	p.o.
Group IV—(NSO_2)	NSO (2 mL/ kg BW)	p.o.
Group V—(NSO_4)	NSO (4 mL/ kg BW)	p.o.

Abbreviations: BW, body weight; i.p, intraperitoneally; NSO, Nigella sativa oil; p.o, orally.

**Table 2:** Experimental design of Freund’s adjuvant-induced sub-acute inflammation.

Groups/Abbrev.	Administrated Substances/Dose/Time	Route
Group I—control group (CTRL)	Normal saline solution	p.o.
Group II—positive control group (CTRL+)	Diclofenac sodium (5 mg/kg BW)	i.p
Group III—(NSO_Prev)	NSO (4 mL/ kg BW)/ 7 days before FA	p.o.
Group IV—(NSO_Treat)	NSO (4 mL/ kg BW)/ 7 days after FA	p.o.
Group V—(NSO_Adj)	NSO (4 mL/ kg BW) + diclofenac sodium (5 mg/kg -BW) / 7 days after FA	p.o./ i.p

Abbreviations: BW, body weight; FA, Freund’s adjuvant; i.p, intraperitoneally; NSO, Nigella sativa oil; p.o, orally.

**Table 3:** Gas chromatography coupled with mass spectroscopy (GS-MS) chemical composition of volatile compounds identified in NSO.

Compounds	RT (min)	Concentration (% from Total Peaks Area)
α-Thujene	7.7	12.02
α-Pinene	7.948	2.49
4(10)-Thujene (Sabinene)	9.325	1.02
β-Pinene	9.501	2.39
(+)-4-Carene	10.949	0.76
*p*-Cymene	11.236	39.72
D-Limonene	11.407	1.61
Sabinene hydrate	14.837	3.72
1-Terpinen-4-ol	17.236	0.35
Thymoquinone	19.806	29.85
Thymol	21.562	2.35
α-Longipinene	23.46	0.66
D-longifolene (Junipene)	25.549	3.05

Abbreviations: min, minutes; RT, retention time.

**Table 4:** The effect of NSO on paw volume in the acute inflammation.

Time Point	Paw Volume (ml) Mean ± SD and Inhibition Percentage (%)				
	CTRL	CTRL+	NSO_1	NSO_2	NSO_4
0 min	2.12 ± 0.31	2.47 ± 0.18	2.42 ± 0.27	2.59 ± 0.3	2.54 ± 0.18
90 min	3.67 ± 0.26	2.65 ± 0.26 ^***^ (27.35%)	3.29 ± 0.31 ^*^ (9.95%)	3.03 ± 0.35 ^***^ (17.11%)	2.90 ± 0.26 ^***^ (20.78%)
180 min	4.04 ± 0.35	2.68 ± 0.41 ^***^ (32.99%)	3.46 ± 0.32 ^*^ (13.86%)	3.43 ± 0.58 ^*^ (15.22%)	3.36 ± 0.43 ^**^ (16.27%)
270 min	4.12 ± 0.20	2.97 ± 0.38 ^***^ (27.67%)	3.83 ± 0.81 (7.26%)	3.43 ± 0.46^*^ (16.76%)	3.42 ± 0.39 ^*^ (17.12%)
360 min	4.00 ± 0.28	3.36 ± 0.47 ^*^ (15.99%)	3.64 ± 0.46 (8.44%)	3.26 ± 0.39 ^**^ (18.25%)	3.05 ± 0.28 ^***^ (23.41%)

Values are presented as mean±SD (*n* = 10), * *p* < 0.05, ** *p* < 0.01, *** *p* < 0.001 significantly different from the control group.

**Table 5:** Antinociceptive effect of NSO on acute inflammation.

Analgesy-Meter (g)/Time	CTRL	CTRL+	NSO_1	NSO_2	NSO_4
0 min	7.1 ± 1.47	8.1 ± 1.68	7.4 ± 3.4	6.1 ± 2.64	6.3 ± 2.00
90 min	6.1 ± 1.35	10.2 ± 2.93 ***	5.9 ± 0.99	5.9 ± 1.91	6.5 ± 2.22
180 min	5.4 ± 1.50	8.6 ± 0.90 **	5.1 ± 2.07	3.8 ± 2.04	5.1 ± 2.46
270 min	6.2 ± 1.51	8.1 ± 1.47 *	5.3 ± 1.70	4.3 ± 2.05 *	4.6 ± 0.96
360 min	5.7 ± 1.39	8.5 ± 1.25 **	3.9 ± 1.85	3.3 ± 1.41 *	4.4 ± 2.54

Values are presented as mean ± SD (*n* = 10), * *p* < 0.05, ** *p* < 0.01, *** *p* < 0.001 significantly different from the control group.

**Table 6:** Anti-inflammatory effect of NSO on sub-acute inflammation.

Time (h)	Paw Volume (ml) Mean ± SD and Inhibition Percentage (%)				
	CTRL	CTRL+	NSO_Prev	NSO_Treat	NSO_Adj
0 h	3.5 ± 0.30	3.9 ± 0.44	3.3 ± 0.64	3.5 ± 0.43	3.4 ± 0.54
24 h	7.4 ± 1.07	6.1 ± 0.99 * (17.1%)	6.3 ± 1.05 (16.7%)	6.5 ± 1.08 (9.7%)	6.6 ± 0.84 (10.8%)
72 h	6.3 ± 0.94	5.4 ± 0.84 (15.7%)	6.5 ± 0.70 -	7.4 ± 1.34 -	5.1 ± 0.56 * (20.1%)
168 h	5.8 ± 1.03	4.7 ± 0.67 * (16.7%)	5.3 ± 1.05 (3.7%)	5.9 ± 1.19 -	4.5 ± 0.52 * (19.2%)

Values are presented as mean ± SD (*n* = 10); * *p* < 0.05 significantly different from the control group.

**Table 7:** Analgesic effect of NSO on sub-acute inflammation.

Analgesy-Meter (g)/Time	CTRL	CTRL+	NSO_Prev	NSO_Treat	NSO_Adj
1 h	6.9 ± 2.84	7.2 ± 2.69	4.7 ± 1.05	4.8 ± 0.78	5.50 ± 0.84
24 h	4.4 ± 0.96	6.4 ± 1.57	5.7 ± 2.21	5.1 ± 2.33	8.3 ± 2.00 ***
72 h	6.6 ± 0.84	8.5 ± 1.58	5.6 ± 2.98	6.3 ± 2.00	10.0 ± 1.69 ***
168 h	4.8 ± 0.78	8.5 ± 1.43 ***	7.4 ± 1.95 **	6.2 ± 1.98 ***	10.6 ± 1.34 ***

Values are presented as mean ± SD (*n* = 10); ** *p* < 0.01, *** *p* < 0.001 significantly different from the control group.

**Table 8:** Analgesic effect of NSO on the sub-acute inflammation rat model.

Reaction Time Hot Plate (sec)/Time	CTRL	CTRL+	NSO_Prev	NSO_Treat	NSO_Adj
1 h	4.4 ± 0.69	6.1 ± 1.66	8.8 ± 4.26 **	5.4 ± 0.84	7.3 ± 1.70 *
24 h	5.2 ± 1.54	8.3 ± 2.05 *	7.9 ± 3.17	7.8 ± 2.04	7.6 ± 2.22
72 h	6.0 ± 1.05	7.7 ± 1.94	6.4 ± 1.42	6.8 ± 1.47	8.3 ± 2.40 *
168 h	3.2 ± 1.13	8.1 ± 1.85 ***	4.5 ± 0.97	4.4 ± 1.07	8.9 ± 2.60 ***

Values are presented as mean ± SD (*n* = 10); * *p* < 0.05, ** *p* < 0.01, *** *p* < 0.001 significantly different from the control group.
